# Superhydrophobicity, Photocatalytic Self-Cleaning and Biocidal Activity Combined in a Siloxane-ZnO Composite for the Protection of Limestone

**DOI:** 10.3390/biomimetics9090573

**Published:** 2024-09-22

**Authors:** Panagiotis N. Manoudis, Ioannis Zuburtikudis, Georgios Konstantopoulos, Hadil Abu Khalifeh, Christine Kottaridi, Ioannis Karapanagiotis

**Affiliations:** 1 Lysis Consulting P.C., 55534 Thessaloniki, Greece; pan.manoudis@gmail.com; 2 Department of Chemical Engineering, Abu Dhabi University (ADU), Abu Dhabi P.O. Box 59911, United Arab Emirates; ioannis.zuburtikudis@adu.ac.ae (I.Z.); hadil.abukhalifeh@adu.ac.ae (H.A.K.); 3 School of Biology, Aristotle University of Thessaloniki, 54124 Thessaloniki, Greece; konstanto@bio.auth.gr (G.K.); ckottaridi@bio.auth.gr (C.K.); 4 School of Chemistry, Aristotle University of Thessaloniki, 54124 Thessaloniki, Greece

**Keywords:** superhydrophobic, water repellent, photocatalytic, biocide, antibacterial, zinc oxide, siloxane, silane, limestone, cultural heritage, coating

## Abstract

The erosion phenomena of the natural stone in cultural heritage are induced by various sources. Consequently, the development of multifunctional protective materials that combine two or more useful properties is an effective strategy in addressing the synergistic effects of various erosion mechanisms. A multifunctional coating, consisting of a silane-based precursor and zinc oxide (ZnO) nanoparticles (NPs), is produced and tested for the protection of limestone. The hybrid coating combines the following three properties: superhydrophobicity, including water-repellency, photocatalytic self-cleaning and biocidal activity. The relative concentration of the NPs (0.8% w/w), used for the suggested composite coating, is carefully selected according to wetting studies, colourimetric measurements and durability (tape peeling) tests. The non-wetting state is evidenced on the surface of the composite coating by the large contact angle of water drops (≈153°) and the small contact angle hysteresis (≈5°), which gives rise to a physical self-cleaning scenario (lotus effect). The photocatalytic chemical self-cleaning is shown with the removal of methylene blue, induced by UV-A radiation. Moreover, it is shown that the suggested coating hinders the incubation of *E. coli* and *S. aureus,* as the inhibitions are 94.8 and 99.9%, respectively. Finally, preliminary studies reveal the chemical stability of the suggested coating.

## 1. Introduction

According to the International Union of Pure and Applied Chemistry (IUPAC), biomimetic surfaces with extreme wetting properties, ranging from superhydrophilicity to superhydrophobicity, are among the top 10 emerging technologies in Chemistry in 2021 [1] due to their numerous potential applications, as described in several review articles [2,3,4,5]. In the area of stone heritage protection, lotus-like, superhydrophobic and water-repellent coatings, defined by a large static contact of water drops (CA > 150°) and a small contact angle hysteresis (CAH < 10°), can be extremely useful in mitigating water-induced degradation phenomena [6]. Typically, the superhydrophobic coatings designed for stone protection are nanocomposites, consisting of polysiloxane (organosilicon) networks and engineered nanoparticles (NPs) [6]. Polysiloxane materials, which originate from silane/siloxane precursors, are selected, as they are recommended for stone heritage consolidation and conservation, with tetraethyl orthosilicate (TEOS) being the most prominent example [7]. NPs are added to the precursor solution and the dispersion is deposited on the target stone. As the silane/siloxane small molecules undergo condensation polymerisation, NP clusters are trapped within the matrix, giving rise to a hierarchically rough surface that mimics the famous structure of the lotus leaf [8,9].

Silicon oxide (SiO_2_) NPs are of low cost, commercially available in various sizes and forms, chemically stable and compatible with polysiloxanes and are therefore very often selected as additives to conservation products to achieve the non-wetting state on natural stone [10,11,12,13,14,15,16]. Calcium hydroxide (Ca(OH)_2_) and calcium carbonate (CaCO_3_) NPs are produced and used because of their chemical compatibility with calcareous stones, which are often used in heritage buildings [17,18]. Other NPs, such as zinc oxide (ZnO) NPs, have been rarely used as additives to polymer-based coatings to achieve superhydrophobicity on natural stone [19,20]. Helmi and Hefni produced a superhydrophobic methyltrimethoxysilane-ZnO composite material, which was deposited on sandstone [19]. In another study, Hefni used a mixture of a fluoropolymer and ZnO NPs to induce superhydrophobicity to treated quartzite blocks [20]. Superhydrophobicity is typically accompanied by a physical self-cleaning mechanism that is based on the rolling motion of water drops carrying away contaminants (lotus effect) [21].

The chemical self-cleaning mechanism is totally different, as it is based on the photocatalytic activity of semiconducting materials, which is activated in the presence of UV radiation and leads to the chemical decomposition of organic pollutants [22]. Following this strategy, titanium oxide (TiO_2_) NPs, the most studied photocatalytic nanomaterial, have been tested to keep built heritage free of contaminants through photocatalysis [23,24,25,26,27]. ZnO is an alternative photocatalyst that exhibits characteristics similar to those of TiO_2_ and has gained increasing popularity for its use in many applications, due to its low cost and biocompatibility [28]. However, only a few studies have investigated the photocatalytic properties of ZnO NPs or modified ZnO NPs for the protection and conservation of natural stone [29,30,31,32]. These studies are briefly reviewed below.

Tokarský et al. produced composite coatings consisting of polysiloxane and ZnO NPs, which were applied to sandstone [29]. The photocatalytic, self-cleaning of methylene blue was demonstrated. However, superhydrophobicity was not achieved, as the maximum CA reported was 128° [29]. In another study [30], the commercially available “Tecnadis Aquashield Forte” medium was used as a host matrix for two nanostructured photocatalysts of TiO_2_-ZnO (50/50 and 10/90) NPs. Dispersions were deposited on lime mortars, limestone, sandstone and granite. Photocatalytic activity was determined by the monitoring of the NO_X_ abatement of the treated specimens. The TiO_2_-ZnO nanoparticles were proven to be more photocatalytically active under solar light than raw TiO_2_ and ZnO materials. Hydrophobicity was obtained on the treated stones, but the threshold of superhydrophobicity (CA = 150°) was not reached [30]. In another report, ZnO NPs, doped with zirconium dioxide (ZrO_2_), were embedded within the matrix of polydimethylsiloxane (PDMS) and the composite material was deposited onto Lecce stone, bricks and marble [31]. The nanocomposite coating showed a moderate hydrophobic character and exhibited a significant photocatalytic, self-cleaning effect based on its ability to discolour methylene blue [31]. ZnO NPs properly doped with bismuth oxide (Bi_2_O_3_) were embedded separately into two commercially available ceramic-based media [32]. The photocatalytic effectiveness of the NPs was shown through a NOx reduction test, which was conducted in a laminar flow reactor. Several suspensions were prepared using different ratios of the aforementioned materials and were applied on sandstone, limestone and granite and superhydrophobicity was achieved in some of the above NP–medium-stone combinations [32].

Apart from its chemical self-cleaning property, ZnO also shows biocidal activity, which is extremely useful for the conservation of heritage stone. Hence, ZnO NPs have been deposited onto various natural stones, offering protection against the growth of microorganisms [33,34,35,36,37]. In these studies, ZnO NPs were directly deposited onto stones without using any low surface energy (organic, fluoro-organic or organosilicon) binders and, therefore, achieving superhydrophobicity was not included in the goals of these investigations [33,34,35,36,37]. Moreover, the photocatalytic self-cleaning effect was not investigated [33,34,35,36,37]. ZnO nanopowders were dispersed in the polysiloxane consolidants by Ditaranto et al. [38], but the wetting of the photocatalytic properties of the composite coating were not investigated. Only biological tests were conducted, giving evidence of the remarkable biostatic activity of the tested nanocomposites [38].

In three studies related to stone heritage conservation, the biocidal activity of ZnO (or functionalised ZnO) NPs was investigated in parallel with its chemical self-cleaning or wetting properties [39,40,41]. In particular, the photocatalytic and antifungal activity of Zn-doped magnesium oxide (MgO) NPs were evaluated in comparison to single ZnO and MgO NPs [39]. Colloidal suspensions were deposited on calcareous stones and showed that the hybrid NPs were superior to the pure MgO or ZnO NPs in both photocatalytic and antifungal tests [39]. In another study, ZnO NPs were embedded in an acrylic resin and the composite material was deposited on marble [40]. It was shown that the conservation material improved the durability of the stone surface in resisting fungal attacks when subjected to inoculums containing *Aspergillus niger* and *Penicillium* sp. Moreover, the composite coating showed enhanced hydrophobicity, as evidenced by the large CA of water drops (=140°) [40]. Finally, the biocidal effectiveness of ZnO and ZnTiO_3_ NPs were shown in another study [41]. The NPs were blended with acrylic and fluorinated polymers and the composite materials were applied on marble. However, very low CAs (<100°) were reported on the surfaces of the composite coatings [41].

In the present study, a multifunctional coating is produced that combines the following three properties: superhydrophobicity, including water-repellency and physical self-cleaning properties, photocatalytic (chemical) self-cleaning and biocidal activity. The three properties are very useful for the preservation of stone heritage by mitigating water-induced degradation mechanisms (through superhydrophobicity and water repellency), to tackle the dirt and dust soiling problem (through physical and chemical self-cleaning activities) and to protect natural stone from biodeterioration (through biocidal activity). The coating is prepared using a commercially available aqueous silane-based system and ZnO NPs and it is applied on limestone. To the best of our knowledge this is the first investigation that reports the production of a material that combines the three aforementioned properties and is produced for the protection of limestone. First, the effects of the NP concentration on the (i) surface morphology, (ii) wetting properties and (iii) colour of treated limestone are investigated. The results of the wettability and colourimetric studies are used to select the optimum ZnO NP concentration which is furthermore supported by the results of durability (tape peeling) tests. It is shown that the composite coating, with the selected NP concentration, removes methylene blue from the surface of coated limestone under the effect of UV-A radiation and hinders the incubation of *E. coli* and *S. aureus*. Finally, preliminary studies reveal the chemical stability of the selected composite coating.

## 2. Experimental

### 2.1. Materials

Protectosil SC 30 (Evonik, Essen, Germany) is an aqueous silane-based system that is free from volatile organic components (VOCs) and is recommended to be used for the protection of natural stone. Zinc oxide (ZnO) NPs (<100 nm particle size) were purchased from Aldrich (St. Louis, MI, USA). Blocks (5 × 5 × 2 cm) of limestone, originating from Crete (Greece) were obtained from K-Stones (Athens, Greece). Distilled water and diiodomethane (Acros Organics, Geel, Belgium) were used to study the wetting properties of the coatings. Aqueous hydrochloric acid solution (HCl) and sodium hydroxide (NaOH) were purchased from Penta Chemicals (Prague, Czech Republic) and they were used to prepare drops of different pH. Methylene blue was obtained from Merck (Rahway, NJ, USA) and it was used to study the physical and chemical (photocatalytic) self-cleaning phenomena.

### 2.2. Coating Preparation and Deposition

ZnO NPs were dispersed into the as-received Protectosil SC 30 product. Dispersions with NP concentrations of 0.5, 0.8, 1.0 and 2.0% w/w were prepared. Dispersions were stirred vigorously for 20 min using an overhead stirrer (Nanostar 7.5, IKA-Werke, Staufen, Germany) equipped with a four-bladed propeller and operated at 400 rpm. Dispersions (2 mL) were cast on the surfaces of the limestone specimens using a pipette. For comparison, Protectosil SC 30 without ZnO NPs was also deposited on limestone. Coated limestone samples were placed in a lab oven at 70 °C for 2 h and were stored in the room conditions for 24 h. This thermal treatment was carried out to accelerate the curing of the coating, before conducting the subsequent experiments of this laboratory study. In real-life applications, coated limestone will be exposed to atmospheric conditions (i.e., lower temperatures) and, therefore, prolonged periods will be necessary for curing.

The coatings that were prepared using only Protectosil (without ZnO NPs) are named hereafter as P0. The coatings that were prepared using Protectosil and ZnO NPs with concentrations of 0.5, 0.8, 1.0 and 2.0% w/w are named P0.5, P0.8, P1.0 and P2.0, respectively. Therefore, the number that follows the letter “P” indicates the relative concentration of ZnO NPs in the dispersion that was used to prepare the coating.

### 2.3. Characterisation and Sample Treatments

Contact angles and the contact angle hysteresis of water drops (8 μL) were measured using the ImageJ software, which was also applied to study drops of diiodomethane and aqueous solutions, which were prepared using HCl or NaOH and corresponded to a pH range from 1 to 14. Drops of different pH were placed to test the chemical stability of the P0.8 coating. At least three drops were placed on different areas of coated limestone specimens and the average values were calculated.

Scanning electron microscopy (SEM; TM3000, Hitachi, Tokyo, Japan) was employed to study surface morphologies. Colourimetric measurements were performed using a PCE-CSM 1 spectrophotometer (PCE instruments, Hamble-le-Rice, UK) and the results were evaluated using the $L^{*}$, $a^{*}$, $b^{*}$ coordinates of the CIE 1976 scale. The physical self-cleaning scenario was shown with methylene blue particles, which were used to deliberately contaminate the surface of coated limestone. The tape peeling test was carried out using Scotch Tape 600 (3 M) according to the ASTM D3359 97 standard test (method A) [42]. The coated surfaces of limestone blocks were subjected to successive attachment–detachment cycles. The test was terminated after 25 cycles.

A homemade chamber equipped with a 300 W Osram Ultra Vitalux light (UV-A component) was employed to study the photocatalytic decolourisation (chemical self-cleaning) of methylene blue on coated limestone. Five drops (2 mL) of aqueous solution of methylene blue (0.1% w/w) were placed on coated and pristine limestone specimens, which were then exposed to the UV light for a total period of 36 h. The same chamber, which was employed for the photocatalytic experiments, was furthermore used to study the effect of the UV light on the wetting properties and colour of the P0.8 coating, i.e., the chemical stability of the P0.8 coating under UV light was tested. The total period of treatment was 36 h.

The gram-negative *Escherichia coli* (NCIMB #12210) and the gram-positive *Staphylococcus aureus* (NCIMB #8625) were used for the antimicrobial testing. Both species were cultivated in autoclaved nutrient broth (NB) media. Limestone specimens treated with the P0 and P0.8 coatings were placed separately in sterilised containers and 400 μL of each culture was inoculated on the surface of each sample. The specimens were incubated overnight at room temperature in a saturated environment. The following day, the specimens and the membranes were washed with 10 mL of autoclaved 0.9% NaCl for 30 min under agitation. This suspension was used for serial sub-decimal dilutions, which were plated on Petri dishes containing nutrient agar (NA) and incubated overnight at 37 °C. For reference, 400 μL of the original culture was inoculated in 10 mL 0.9% NaCl in order to imitate the dilution that occurs during the washing step. This suspension was also used for serial sub-decimal dilutions and plating in NA petri dishes. After incubation, the colonies on each dish were counted and the cfu/mL that could be retrieved from each specimen was calculated and compared to the cfu/mL of the reference.

## 3. Results and Discussion

### 3.1. Surface Morphology and Wetting Properties vs. NP Concentration

**Figure 1:**
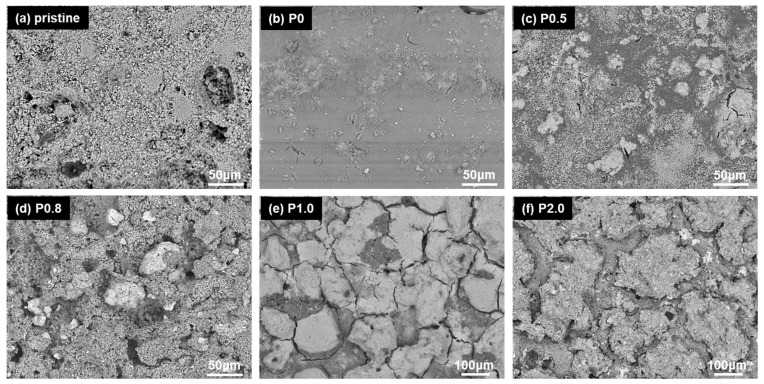
SEM images of (**a**) uncoated limestone, (**b**) limestone coated with only Protectosil without using ZnO NPs (P0) and limestones coated with Protectosil blended with (**c**) 0.5% (P0.5), (**d**) 0.8% (P0.8), (**e**) 1.0% (P1.0) and (**f**) 2.0% (P2.0) w/w ZnO NPs.

Figure 1c–f shows the evolution of the surface morphology of coated limestone as a function of the ZnO NP concentration. The pristine limestone is included in the SEM image of Figure 1a, which shows that the stone surface is rough, bearing large pores. The application of the P0 coating (no NPs) results in a smoother surface as the stone pores are filled by the conservation material (Figure 1b). Adding NPs to the protective coating results in different structures that depend on the NP concentration (Figure 1c–f). NPs form protruded microscale clusters which, in the case of the P0.5 coating, are separated by smooth coated areas (Figure 1c). As the NP concentration increases, the protruded clusters become bigger and coalesce, resulting progressively in a highly rough surface (Figure 1d–f). The scenario revealed in Figure 1 is in agreement with previously published reports that investigated the evolution of surface structures of various nanocomposite coatings, consisting of polymers and SiO_2_ NPs [6,8,9,12].

To further show the effect of the ZnO NPs on the surface morphology of the coatings, EDS elemental maps of Zn were acquired from the surface of a limestone specimen, which was treated with the P2.0 coating. An elemental map and the corresponding SEM image are provided in Figure S1a,b of the Supplementary File. It is shown that the concentration of Zn is elevated in the regions of the protruded microscale clusters, suggesting that the ZnO NPs are responsible for the formation of the protrusions and, therefore, the evolution of the highly rough surface. To show that the recorded Zn originates from the added NPs, EDS elemental maps of Zn and SEM images were acquired from a limestone sample treated with the P0 coating (Figure S1c,d) and an uncoated limestone specimen (Figure S1e,f). The results show that in both P0 and uncoated samples, Zn was not detected.

**Figure 2:**
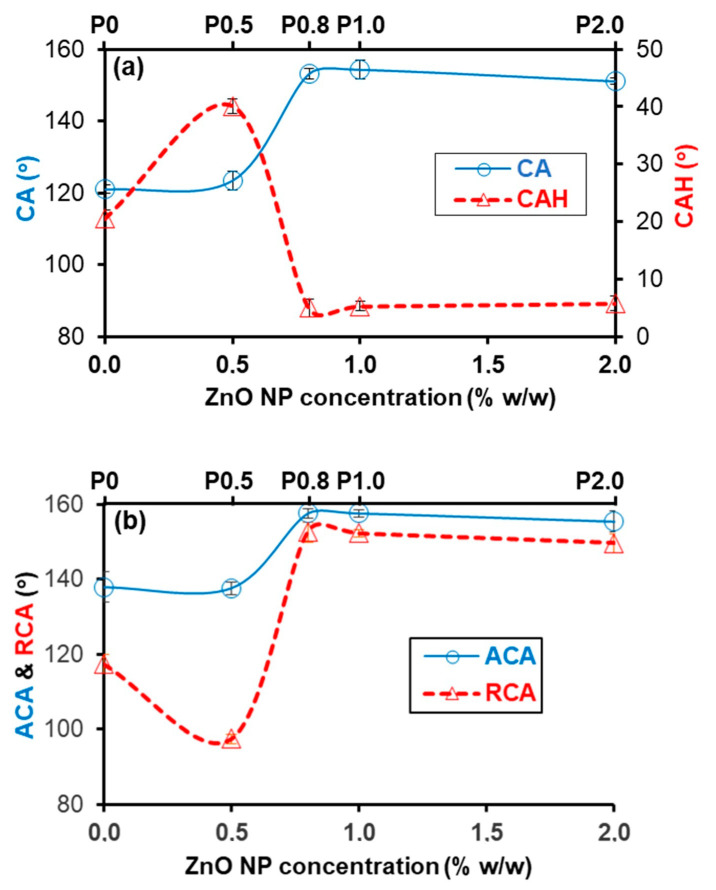
(**a**) Contact angle (CA) and contact angle hysteresis (CAH) of water drops on coated limestone samples vs. the ZnO NP concentration. (**b**) Advancing (ACA) and receding (RCA) contact angles of water drops on coated limestone samples vs. the ZnO NP concentration.

Figure 2a shows contact angle (CA) and contact angle hysteresis (CAH) results on coated limestone. The P0 coating corresponds to a CA of 121.1 ± 1.2°. It should be noted that the surface of the P0 coating is not perfectly smooth (Figure 1b), as its morphology is affected by the highly rough underlying limestone substrate. Roughness gives rise to the measured CA. Indeed, the P0 coating gave a CA of 113.8 ± 1.3° when it was deposited on glass, which is much lower than the 121.1 ± 1.2° value reported in Figure 2a for the P0 coating on rough limestone [6,9]. According to the results of Figure 2a, adding a small concentration (0.5% w/w) of NPs does not have any effect on CA, but it results in a rapid increase in CAH (P0.5 coating). A slightly higher NP concentration (0.8% w/w) causes a rapid increase in CA and a major decrease in CAH. The further increase in the NP concentration to 1 and 2% w/w does not have any practical effect on the contact angles that are roughly stable, corresponding to a very large CA (around 153°) and a very small CAH (around 5°). Consequently, coatings which were prepared using high NP concentration (≥0.8% w/w) show superhydrophobic (CA > 150°) and water repellent properties (CAH < 10°), similar to those reported for the leaves of lotus [21].

The origin of the CAH variation, reported in Figure 2a, is elucidated in Figure 2b. CAH is defined as the difference between the advancing (ACA) and the receding (RCA) contact angles. The P0 and P0.5 coatings correspond to comparable ACAs. As the NP concentration increases from 0.5 to 0.8% w/w, ACA increases rapidly. The further increase in the NP concentration does not affect ACA, which is large and roughly constant for coatings prepared using NP concentration ≥ 0.8% w/w. The behaviour of RCA is more complicated. As the NP concentration increases, RCA initially decreases (as the NP concentration increases from 0 to 0.5% w/w), then it increases abruptly (as the NP concentration increases from 0.5 to 0.8% w/w) and, finally, RCA becomes constant at elevated NP concentration (≥0.8% w/w). Notably, as shown in Figure 2b at NP concentration ≥ 0.8% w/w, ACA and RCA become comparable, leading to a very small CAH, which is reported in Figure 2a.

The increase in CA with NP concentration leading to superhydrophobic Protectosil-ZnO composites (Figure 2a) has been reported several times for various polysiloxane-SiO_2_ composites deposited onto natural stones [6,8,9,12]. However, the delicate trend of CAH, shown in Figure 2a, has been rarely reported, and only for the polysiloxane-SiO_2_ composite coatings that were deposited on glass. In particular, Manoudis et al. [8] and Karapanagiotis and Manoudis [9] observed an increase, followed by an abrupt decrease in CAH with NP concentration for polyalkylsiloxane-SiO_2_ coatings. Basu et al. reported a similar behaviour of CAH for methyltriethoxysilane-hydrophobic SiO_2_ composites [43], whereas Baba et al. noticed the same variation in CAH for polystyrene–hydrophobic SiO_2_ composites [44].

The reported wetting properties of the coatings in Figure 2 can be explained by their surface structures (Figure 1) in light of previously published studies [8,9]. The protrusions that are formed on the P0.5 surface are separated by large, relatively smooth areas (Figure 1c). A water drop can fill these large, smooth interspaces that exist among the protrusions, which can therefore act as obstacles (pinning sites) for the motion of the drop, thus resulting in an increase in CAH. Therefore, the Wenzel scenario (or a mixed Wenzel and Cassie–Baxter state) can be taken into account to explain the wetting properties of the P0.5 surface, as has been previously suggested for polysiloxane-SiO_2_ composite coatings on glass [8,9]. At a higher NP concentration (≥0.8% w/w), the protruded aggregates become bigger (Figure 1d–f), eliminating the smooth interspaces and, therefore, the pinning sites, thus leading to water repellency, which is evidenced by the small CAH (Figure 2). A water drop can stay suspended on top of the protruded aggregates and, therefore, the non-sticking, Cassie–Baxter scenario is rationalised. Although the surface morphologies of the P0.8, P1.0 and P2.0 coatings show some differences (Figure 1d–f), their wetting properties are comparable (Figure 2), exhibiting superhydrophobicity and water repellency. However, the three superhydrophobic coatings induce different colour changes to limestone as is discussed later.

**Figure 3:**
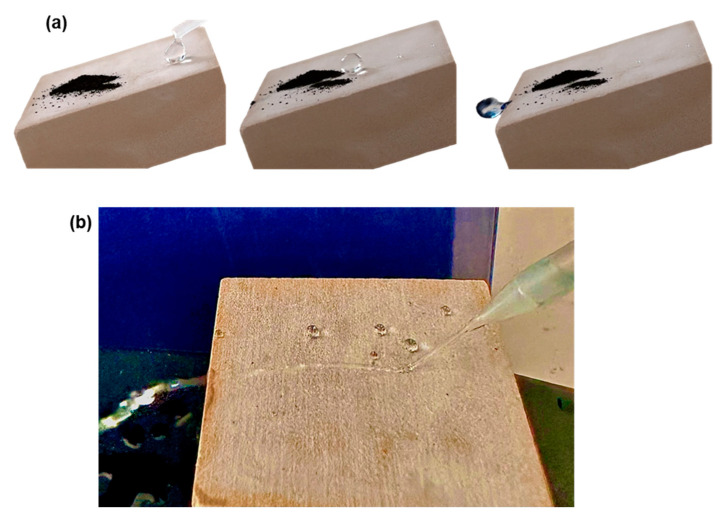
(**a**) Successive snapshots showing the physical self-cleaning scenario (lotus effect) on the contaminated limestone that was treated with the P0.8 coating. (**b**) Water jet flow is repelled by the coated (P0.8) limestone sample.

The water repellent properties of the P0.8 coating are highlighted by Figure 3, Video S1 (Supplementary File) and the result of the Owens–Wendt method, as follows. In the successive snapshots of Figure 3a it is shown that methylene blue particles, which were deliberately placed on a tilted limestone sample coated with P0.8, were easily removed by water drops. This is the physical self-cleaning mechanism that was first revealed in the surface of the lotus leaf [21]. Figure 3b shows that the water jet flow is reflected by the repellent character of the P0.8 coating. The force per unit length that causes the drop to move along a surface, is given by [45,46]

(1) $$F=\gamma_{L}(cosRCA-cosACA)$$

where $\gamma_{L}$ is the surface tension of the liquid. Taking into account the values of ACA and RCA, which are provided in Figure 2b for the P0 and P0.8 coatings, it has been calculated that it takes about eight times as much force to move a drop on the surface of the P0 coating than on the surface of the P0.8 coating.

The non-sticking properties of the P0.8 coating are further demonstrated in Video S1 (Supplementary File). The water drop does not adhere to the surface of the P0.8 coating. Instead, it remains attached to the needle following its motion along the surface of coated limestone.

The repellent character of the P0.8 coating originates from its special surface structure, which corresponds to a very low apparent surface energy. The latter can be calculated following the Owens–Wendt method [47]:

(2) $$\gamma_{L}\left(1+cosCA\right)=2\left[\sqrt{\gamma_{L}^{d}\gamma_{S}^{d}}+\sqrt{\gamma_{L}^{p}\gamma_{S}^{p}}\right]$$

(3) $$\gamma_{S}=\gamma_{S}^{d}+\gamma_{S}^{p}$$

where $\gamma_{L}^{d}$ and $\gamma_{L}^{p}$ are the dispersive and polar components of the surface free energy of the liquid ($\gamma_{L}$) and $\gamma_{S}^{d}$ and $\gamma_{S}^{p}$ are the corresponding components of the surface free energy of the solid ($\gamma_{S}$). Water and diiodomethane drops were placed on the P0.8 coating on limestone and Equation (2) was applied using the values that are included in Table 1. Using Equation (3), it was then calculated that the apparent surface energy of the P0.8 surface is only 2.0 $mJ/m^{2}$. Notably, this result is close to the apparent surface energy (1.1 $mJ/m^{2}$) of the bionic surface, which was prepared by imitating the lotus leaf surface’s microstructure [48].

### 3.2. Selection of NP Concentration

The results in Figure 2 show that the minimum NP concentration that should be added to protective coating to achieve superhydrophobicity and water repellency is 0.8% w/w. The photographs of Figure 3 and Video S1 provide further evidence of the repellent character of the P0.8 coating. In this paragraph it is shown that the results of the colourimetry and the tape peeling tests suggest that the P0.8 coating is a better option for the protection of heritage limestone, compared to the other superhydrophobic composite coatings, i.e., P1.0 and P2.0. The two tests are important for the following reasons. Colourimetry highlights the colour alteration that a conservation product causes to the original stone. Large colour changes in the pristine stone are not acceptable in heritage science. The tape peeling test provides results on the mechanical durability of the coating. It is known that the poor durability of the hierarchical structured, non-wettable surfaces is considered to be the major obstacle towards real life applications [49].

**Figure 4:**
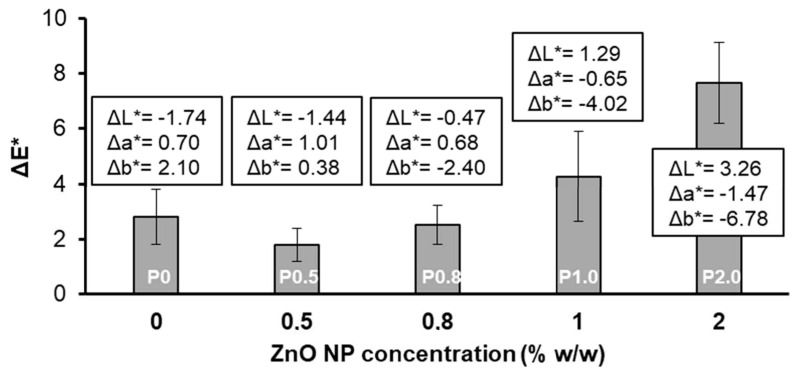
Colour change (${\Delta E}^{*}$) in the coated limestone samples vs. the ZnO NP concentration. The colours of the limestone samples changed due to the application of the coatings. The mean values of the calculated changes in the $L^{*}$, $a^{*}$ and $b^{*}$ components are included in the graph.

The results of the colourimetric measurements are summarised in Figure 4. The total colour changes (${\Delta E}^{*}$) in the coated limestone samples with respect to the colour of the pristine (uncoated) limestone were calculated using the following equation:

(4) $${\Delta E}^{*}=\sqrt{({\Delta L}^{*2}+{\Delta a}^{*2}+{\Delta b}^{*2}}$$

where ${\Delta L}^{*}$, ${\Delta a}^{*}$ and $\Delta b^{*}$ are the changes in the brightness, the red–green component and the yellow–blue component of the CIE 1976 scale, respectively, which are defined as follows: ${\Delta L}^{*}=L_{c}^{*}-L_{u}^{*};\;{{\Delta a}^{*}=a}_{c}^{*}-a_{u}^{*};{\Delta b}^{*}=b_{c}^{*}-b_{u}^{*}$. The “c” and “u” subscripts indicate the coated and uncoated samples, respectively. The mean values of ${\Delta L}^{*}$, ${\Delta a}^{*}$ and $\Delta b^{*}$ are included in Figure 4.

According to the ${\Delta E}^{*}$ results of Figure 4, the coatings with low NP concentrations (P0.5 and P0.8 coatings) or without NPs (P0 coating) had only minor effects on the original colour of limestone, which were hardly detected by the naked eye as ${\Delta E}^{*}$ < 3 [50]. Coatings with higher NP concentrations (P1.0 and P2.0 coatings) induced considerable changes to the aesthetic appearance of limestone, corresponding to ${\Delta E}^{*}$ > 3. A major contribution to the increase in ${\Delta E}^{*}$ with NP concentration originated from the change in ${\Delta L}^{*}$. As the ZnO NPs are whitish, they tend to increase the brightness of treated limestone, resulting progressively in more positive and higher ${\Delta L}^{*}$ values. Another noticeable contribution to the variation in ${\Delta E}^{*}$ with NP concentration came from the change in the $b^{*}$ component. Adding more NPs to the coating resulted in more negative and higher ${\Delta b}^{*}$ absolute values. Notably, other NPs, such as SiO_2_ [12] and hydrophobic CaCO_3_ [18], which were added to siloxane coatings on limestone, gave similar colourimetric effects. Finally, according to the results of Figure 4, the contribution of ${\Delta a}^{*}$ to the overall colour change (${\Delta E}^{*}$) was less important, compared to ${\Delta L}^{*}$ and ${\Delta b}^{*}$.

The results of Figure 4 suggest that the maximum ZnO NP concentration that can be practically used for the preparation of the protective coating is 0.8% w/w. Coatings that were prepared using higher NP concentrations (>0.8% w/w) induced considerable changes to the colour of the pristine limestone, which corresponded to ${\Delta E}^{*}$ > 3.

**Figure 5:**
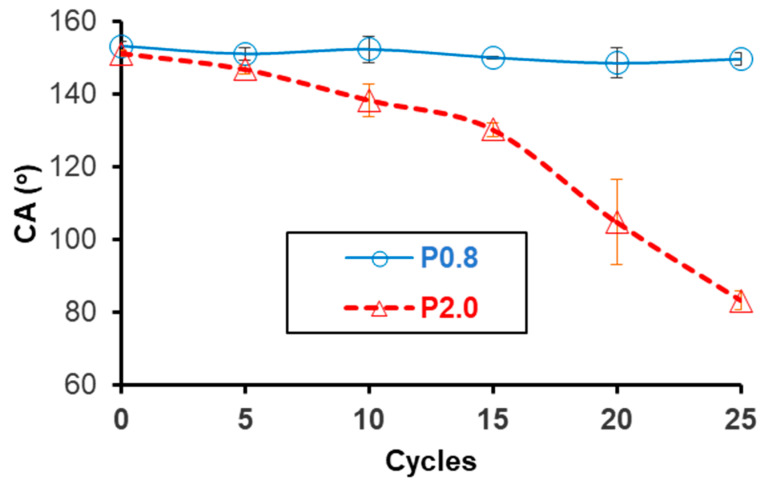
Results for the tape peeling test that was applied on limestone samples treated with the P0.8 and P2.0 coatings. The CA vs. the number of cycles is shown. Twenty-five (25) attachment-detachment cycles were applied in total.

**Figure 6:**
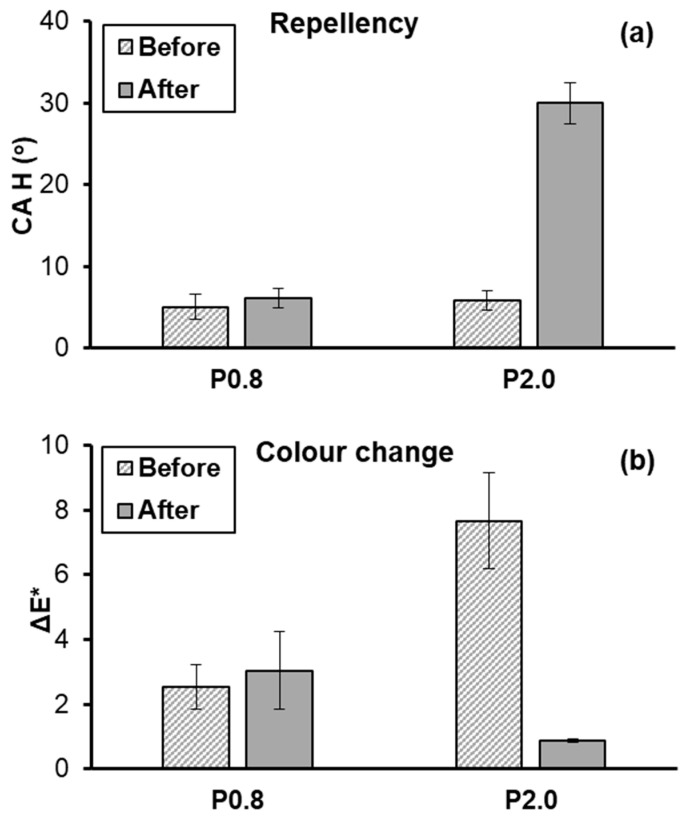
(**a**) Contact angle hysteresis (CAH) and (**b**) colour change (${\Delta E}^{*}$) measured before and after the application of 25 attachment–detachment cycles of the tape peeling test on limestone samples that were treated with the P0.8 and P2.0 coatings. The ΔE* calculations in (**b**) were conducted with respect to the pristine, uncoated limestone.

The tape peeling test was performed to compare the stabilities of superhydrophobic surfaces that were produced using low (0.8% w/w) and high (2.0% w/w) NP concentrations. The CA results are provided in Figure 5 and show the superior durability of the P0.8 coating, compared to the P2.0 coating. After 25 attachment–detachment cycles, the wettability of the P2.0 surface dropped from the superhydrophobic (CA > 150°) to the hydrophilic (CA < 90°) regime. On the contrary, the elevated CA of the P0.8 surface remained practically stable. Figure 6 shows CAH and ${\Delta E}^{*}$ results, which were collected before and after the application of the 25 attachment–detachment cycles. The repellent character of the P0.8 surface was maintained, as CAH was not affected by the application of the tape peeling test, whereas the CAH on the P2.0 surface increased from 5.8 ± 1.2° to 30.0 ± 2.5° (Figure 6a). The ${\Delta E}^{*}$ calculations in Figure 6b were conducted with respect to the original, uncoated limestone. It is seen that the tape peeling test did not have any noticeable effect on the aesthetic appearance of the stone specimen that was treated with the P0.8 coating. The colour change, which was induced to the stone by the deposition of the fresh P0.8 coating, was maintained after the application of the tape peeling test. On the contrary, after the tape peeling test, ${\Delta E}^{*}$ of the specimen that was treated with the P2.0 coating dropped to roughly 0.9, suggesting that the limestone sample almost obtained its original colour measured prior to the deposition of the coating. This result highlights the poor adhesion of the P2.0 coating with the limestone substrate.

In sum, based on the results of Figure 5 and Figure 6, it is determined that the use of a high NP concentration in order to safely achieve the non-wetting state has a negative effect on the stability of composite coating. Consequently, selecting the minimum NP concentration (i.e., 0.8% w/w), which is necessary to achieve superhydrophobicity and water repellency (Figure 2a), is the logical choice to enhance coating’s durability. Overall, the results of the colourimetry (Figure 4) and the tape peeling (Figure 5 and Figure 6) tests suggest that the best superhydrophobic Protectosil-ZnO coating that can be selected for the protection of limestone is the P0.8 coating.

### 3.3. Chemical (Photocatalytic) Self-Cleaning and Biocidal Activity

**Figure 7:**
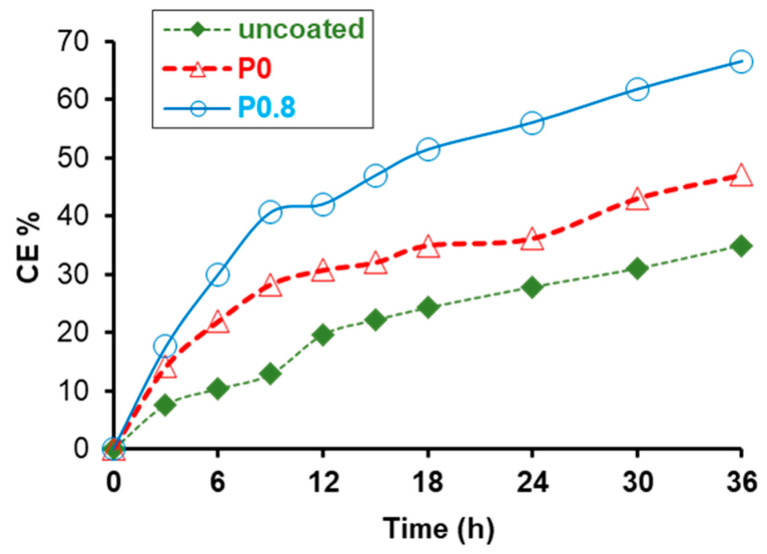
Cleaning efficacies (CE%) of coated (P0 and P0.8) and uncoated limestones that were contaminated with methylene blue were calculated using Equation (5) for various periods of treatment under the UV light.

The physical self-cleaning property of the P0.8 coating, originated by its water repellent character, was discussed and revealed in Figure 2 and Figure 3 and Video S1. The chemical self-cleaning property of the P0.8 coating is shown in Figure 7. Samples coated with superhydrophobic P0.8, hydrophobic P0 and uncoated limestone specimens were contaminated with methylene blue and the removal of the dye under the effect of the UV radiation was measured. Uncoated limestone and limestone coated with P0 were included in the study for comparative purposes to report on the removal of the dye without the involvement of the photocatalytic mechanism. The results in Figure 7 are presented with respect to the relative cleaning efficacy (CE%), which was calculated using the following formula [51,52,53]:

(5) $$CE\%=(\frac{{\Delta E}_{b}^{*}-{\Delta E}_{a}^{*}}{{\Delta E}_{b}^{*}})100$$

where ${\Delta E}_{b}^{*}$ and ${\Delta E}_{a}^{*}$ are the colour changes before and after the UV treatment of the sample stained with methylene blue. Both colour changes were measured with respect to the unstained sample. In the ideal case of the total removal of the stain, ${\Delta E}_{a}^{*}$ becomes 0 and, therefore, the relative cleaning efficacy (CE%) becomes 100. Figure 7 shows that, after 36 h of treatment, a considerable amount of dye was removed from the uncoated limestone, due to the effect of the intensive UV radiation. A greater degree of degradation of methylene blue was achieved on the P0-coated sample, suggesting that the hydrophobic character of Protectosil is capable of promoting the removal of the dye, compared to the uncoated limestone. The same result was reported in a previously published study in which chemical self-cleaning was promoted by the hydrophobic character of a fluoro-siloxane coating on glass [53]. As shown in Figure 7, dye removal was further assisted by ZnO NPs, resulting in even higher values of CE% for the sample treated with the P0.8 coating. From the results of Figure 7, it has been calculated that the relative increase in CE% with respect to the uncoated sample was 35% for the P0-coated sample and 91% for the P0.8-coated sample. Consequently, it is reported that even at the low concentration of 0.8% w/w, the photocatalytic mechanism, which was induced by ZnO, had a major effect on the removal of methylene blue.

**Figure 8:**
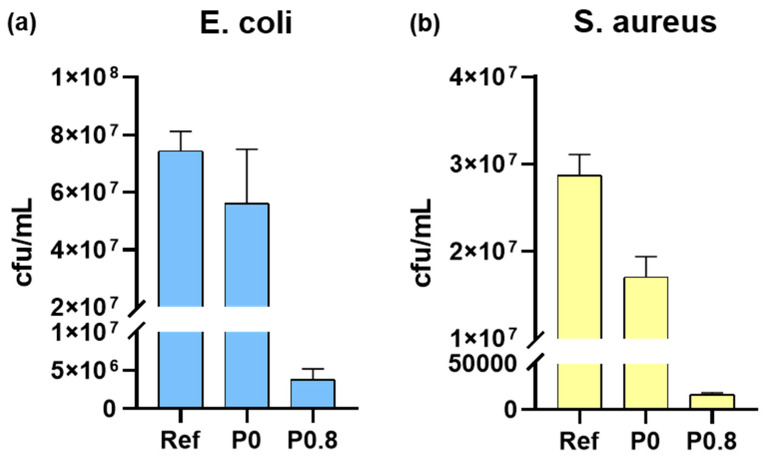
The graphs show the cfu/mL that was retrieved from the limestones coated with P0 and P0.8 and inoculated with (**a**) *E. coli* and (**b**) *S. aureus* liquid cultures, in comparison to the reference (Ref).

The quantitative results of the biocide tests are shown in Figure 8. In particular, the cfu/mL results of the *E. coli* (Figure 8a) and *S. aureus* (Figure 8b) cultures that were retrieved from limestone samples treated with the P0 and P0.8 coatings are shown. To assess the biocidal activity of the two coatings, the results for the reference cultures (Ref) are included in the two graphs. The results show a similar tendency for both bacteria. The incubation of the two cultures is hindered by the P0 coating, which appears to be more effective in the prevention of the growth of *S. aureus*. However, stone protection against the growth of the two micro-organisms is largely enhanced when the P0.8 coating is used. Τhe cfu/mL values for the *E. coli* and *S. aureus* cultures that were retrieved from the limestone specimens coated with P0.8 are much lower compared to the corresponding values reported for limestones coated with P0. Using the results of Figure 8, it was calculated that the samples treated with the P0 coating demonstrated 24.4 and 40.6% inhibition of *E. coli* and *S. aureus*, respectively. The inhibitions against the growth of *E. coli* and *S. aureus* increased to 94.8 and 99.9%, respectively, for the limestone samples treated with the P0.8 coating. Consequently, even at the low concentration of 0.8% w/w, the ZnO NPs were effective in inducing major biocidal activity.

### 3.4. Other Tests

**Figure 9:**
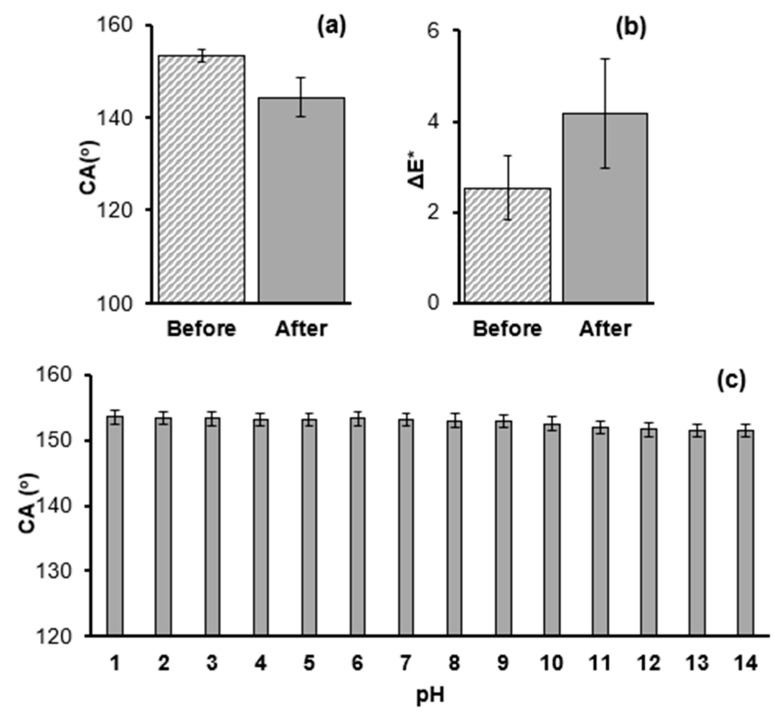
Stability of the P0.8 coating on limestone. (**a**) CA and (**b**) ${\Delta E}^{*}$ results before and after exposing the samples to artificially accelerated UV radiation. (**c**) CA of drops of aqueous solutions vs. the pH of the drop.

Two more tests were carried out to evaluate the stability of the P0.8 coating. Figure 9a,b reveals the effects of the artificially accelerated ageing process that was developed by exposing the samples to intense UV radiation. According to the results of Figure 9a, the UV treatment caused a noticeable decrease in CA on the P0.8 surface, which, however, remained > 140°. Figure 9b shows ${\Delta E}^{*}$ calculations before and after the UV treatment, which were performed with respect to the original, uncoated limestone. Therefore, the ${\Delta E}^{*}$ (=2.53) value reported in Figure 9b before the application of the UV treatment, was reproduced from Figure 4. According to the graph of Figure 9b, the intense UV light caused an increase in ${\Delta E}^{*}$ (=4.18) which, however, remained < 5. Colour changes that fall within a range of ${\Delta E}^{*}$ between three and five are visible to the naked eye, but are usually acceptable in conservation practice [50]. Notably, the major contribution to the increase in ${\Delta E}^{*}$ originated from the change in ${\Delta L}^{*}$ which increased from −0.47 (before UV treatment) to 1.22 (after UV treatment), suggesting that the coating became more whitish. A similar trend was observed in the P0 coating (not included in Figure 9b) as ${\Delta E}^{*}$ increased, mainly due to the change in ${\Delta L}^{*}$. The results of the second test are shown in Figure 9c. Drops that corresponded to a wide range of pH were placed on the P0.8 coating and CAs were measured. It is seen that CA remained stable (>150°), as it was practically not affected by the pH of the drop. Overall, the results of Figure 9 suggest that the P0.8 coating on limestone showed very good chemical stability against the effects of the UV radiation and the pH of the drop.

## 4. Conclusions

The main message of this work is that a multifunctional coating was produced, consisting of a silane-based precursor and zinc oxide (ZnO) nanoparticles (NPs), for the protection of limestone. The coating combines the following properties: (i) superhydrophobicity, including water-repellency and physical self-cleaning properties, (ii) photocatalytic (chemical) self-cleaning activity and (iii) biocidal activity. These three properties are very useful for the preservation of stone heritage to mitigate water-induced degradation mechanisms, to tackle dirt and dust soiling problems and to prevent biodeterioration.

In particular, the effects of the ZnO NP concentration on the surface morphology, wetting properties and colour of treated limestone were investigated. It was shown that NPs form microscale clusters that coalesce as the NP concentration increases, resulting in a rough surface structure. The latter shows superhydrophobic and water repellent properties, which induce the physical self-cleaning scenario. According to the colourimetric results, the maximum NP concentration that can be practically used for the protection of heritage limestone is 0.8% w/w. Notably, this is the minimum NP concentration that should be used to maintain the superhydrophobic and water repellent character of the composite coating. Moreover, the results of the tape peeling test show that keeping the NP concentration as low as possible is preferable for enhancing the mechanical stability of the composite coating. Hence, the coating corresponding to 0.8% w/w NP concentration (P0.8) was selected for further studies. The P0.8 coating showed sufficient chemical self-cleaning, induced by the photocatalytic activity of ZnO, which was activated in the presence of UV radiation. The inhibitions against the growth of *E. coli* and *S. aureus* on limestone coated with P0.8 were 94.8 and 99.9%, respectively. Finally, the P0.8 coating showed very good chemical stability against the effects of the UV radiation and the pH of the drop.

The stability of the P0.8 coating over extended periods in outside and diverse environmental conditions is an important direction for future work. Monitoring the performance of a conservation material under real-life conditions for a prolonged period of time is crucial to fully assess the compatibility and effectiveness of the material [54]. Other practical challenges, such as maintaining consistency during scaled-up production and application and adapting the method to treat heritage stones other than limestone, should be considered in future studies to strengthen and increase the potential for the P0.8 coating to be used in stone conservation practice.

## Figures and Tables

**Table 1:** Dispersive ($\gamma_{L}^{d}$) and polar ($\gamma_{L}^{p}$) components of the surface free energy of the liquid ($\gamma_{L}$) for water and diiodomethane [47]. CAs of water and diiodomethane are included.

Liquid	$\gamma_{L}^{d}$ (mJ m^−2^)	$\gamma_{L}^{p}$ (mJ m^−2^)	$\gamma_{L}$ (mJ m^−2^)	CA (°)
Water	21.8	51.0	72.8	153.2 ± 1.4
Diiodomethane	49.5	1.3	50.8	129.5 ± 2.3

## Data Availability

Dataset available on request from the authors.

## Supplementary Materials

The following supporting information can be downloaded at: https://www.mdpi.com/article/10.3390/biomimetics9090573/s1, Figure S1: SEM images and EDS elemental maps of Zn for the following samples: (a,b) limestone coated with Protectosil blended with 2.0% w/w ZnO NPs (P2.0), (c,d) limestone coated with only Protectosil without using ZnO NPs (P0) and (e,f) uncoated limestone; Video S1: Water drop on P0.8 coating.
