# Supplementary material for: Superhydrophobicity, Photocatalytic Self-Cleaning and Biocidal Activity Combined in a Siloxane-ZnO Composite for the Protection of Limestone
Source: Biomimetics (Basel). 2024 Sep 22;9(9):573. doi: 10.3390/biomimetics9090573 (PMC11429561; doi:10.3390/biomimetics9090573)
Supplement: Supplementary file 1 [file biomimetics-09-00573-s001.zip › Supplementary File.pdf]

SUPPLEMENTARY FILE

Superhydrophobicity, photocatalytic self-cleaning and biocidal activity combined in a siloxane-ZnO composite for the protection of limestone

Panagiotis N. Manoudis[1], Ioannis Zuburtikudis[2], Georgios Konstantopoulos[3], Hadil Abu Khalifeh[2], Christine Kottaridi[3] and Ioannis Karapanagiotis[4*]

[1]Lysis Consulting P.C., 55534 Thessaloniki, Greece
[2]Department of Chemical Engineering, Abu Dhabi University (ADU), P.O. Box 59911, Abu Dhabi, United Arab Emirates
[3]School of Biology, Aristotle University of Thessaloniki, 54124 Thessaloniki, Greece
[4]School of Chemistry, Aristotle University of Thessaloniki, 54124 Thessaloniki, Greece
[*]Correspondence: karapana@chem.auth.gr

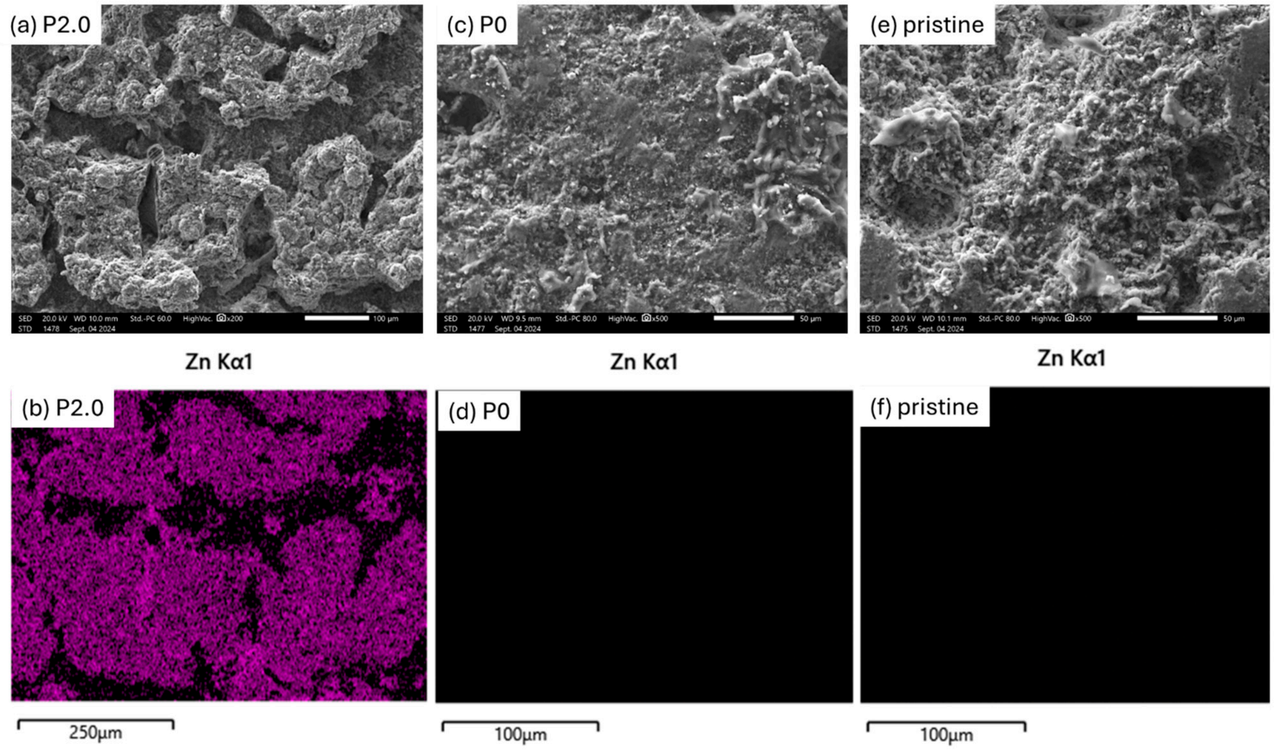

Figure S1. SEM images and EDS elemental maps of Zn for the following samples: (a,b) limestone coated with Protectosil blended with 2.0% w/w ZnO NPs (P2.0), (c,d) limestone coated with only Protectosil without using ZnO NPs (P0) and (e,f) uncoated limestone.
